# Assessing the Electronic Evidence System Needs of Canadian Public Health Professionals: Cross-sectional Study

**DOI:** 10.2196/26503

**Published:** 2021-09-07

**Authors:** Bandna Dhaliwal, Sarah E Neil-Sztramko, Nikita Boston-Fisher, David L Buckeridge, Maureen Dobbins

**Affiliations:** 1 National Collaborating Centre for Methods and Tools McMaster University Hamilton, ON Canada; 2 Department of Health Research Methods, Evidence and Impact McMaster University Hamilton, ON Canada; 3 School of Population and Global Health McGill University Montreal, QC Canada; 4 School of Nursing McMaster University Hamilton, ON Canada

**Keywords:** population surveillance, evidence-informed decision-making, needs assessment, public health, precision public health

## Abstract

**Background:**

True evidence-informed decision-making in public health relies on incorporating evidence from a number of sources in addition to traditional scientific evidence. Lack of access to these types of data as well as ease of use and interpretability of scientific evidence contribute to limited uptake of evidence-informed decision-making in practice. An electronic evidence system that includes multiple sources of evidence and potentially novel computational processing approaches or artificial intelligence holds promise as a solution to overcoming barriers to evidence-informed decision-making in public health.

**Objective:**

This study aims to understand the needs and preferences for an electronic evidence system among public health professionals in Canada.

**Methods:**

An invitation to participate in an anonymous web-based survey was distributed via listservs of 2 Canadian public health organizations in February 2019. Eligible participants were English- or French-speaking individuals currently working in public health. The survey contained both multiple-choice and open-ended questions about the needs and preferences relevant to an electronic evidence system. Quantitative responses were analyzed to explore differences by public health role. Inductive and deductive analysis methods were used to code and interpret the qualitative data. Ethics review was not required by the host institution.

**Results:**

Respondents (N=371) were heterogeneous, spanning organizations, positions, and areas of practice within public health. Nearly all (364/371, 98.1%) respondents indicated that an electronic evidence system would support their work. Respondents had high preferences for local contextual data, research and intervention evidence, and information about human and financial resources. Qualitative analyses identified several concerns, needs, and suggestions for the development of such a system. Concerns ranged from the personal use of such a system to the ability of their organization to use such a system. Recognized needs spanned the different sources of evidence, including local context, research and intervention evidence, and resources and tools. Additional suggestions were identified to improve system usability.

**Conclusions:**

Canadian public health professionals have positive perceptions toward an electronic evidence system that would bring together evidence from the local context, scientific research, and resources. Elements were also identified to increase the usability of an electronic evidence system.

## Introduction

### Background

In the time of growing funding restraints for public health in Canada and across the world, public health professionals and organizations must function efficiently to meet the expanding public health needs. Changes to the funding structure of public health have been underway across Canada for several years [1]. In the province of Quebec, the public health budget was cut by 33% in 2015; cuts of up to 30% were proposed in Ontario in 2019; and more recently, cuts of up to 10% were proposed in Alberta [2-4]. Constraints of public health funding are not limited to Canada; countries such as the United States and England have seen similar trends [5,6]. Exceptions to this trend can occur during times of crisis, including the current COVID-19 (SARS-CoV-2) pandemic, whereby further funding cuts are halted or funding is even increased; however, these exceptions may be limited in duration [7].

In addition to the impacts of restructuring and decreasing funding, the public health sector is challenged to function effectively with the exponential increase in the amount of scientific evidence generated and the local contextual data available, as seen in response to the COVID-19 pandemic. The amount of information available now exceeds the capacity of public health professionals to comprehensively assess, consider, and use in program planning decisions. Given these challenges, there is a need to understand how public health professionals and organizations can meet increasing demands for evidence-informed decision-making with fewer resources [8].

A 2016 scoping review identified 4 factors that were associated with improved efficiency in public health systems: (1) increased financial resources, (2) increased staffing per capita, (3) jurisdictions serving a population of 50,000 to 500,000 people, and (4) evidence-based organizational and administrative features [3]. Although the first 3 factors are controlled at a subnational or federal government level, institutional changes to support evidence-based practices occur at a local level and, therefore, present opportunities for change. Within the category of administrative evidence-based features, one umbrella review identified five high-priority, locally modifiable best practices that contribute to public health system productivity: workforce development, leadership, organizational climate and culture, interorganizational relationships and partnerships, and financial processes [9]. Specifically, access to and free flow of relevant information were identified as factors that can contribute to public health system performance in the short term; this includes ready access to high-quality information and tailored messages for evidence-based decision-making [9].

Evidence-based public health and practice is defined as “the process of integrating science-based interventions with community preferences to improve the health of populations” [10], whereas evidence-informed public health is defined as “using research evidence with public health expertise, resources, and knowledge about community health issues, local context, and political climate to make policy and programming decisions” [11,12]. Using the term *informed* rather than *based* allows for nuances of the decision-making process that are not solely based in research evidence, such as considerations of the political climate and expertise of public health professionals [9,13]. Using evidence to inform program planning decisions increases the likelihood that services with known effectiveness will be delivered and supports the efficient use of human and financial resources. Across Canada, evidence-informed decision-making is becoming a central tenant of public health and is now incorporated into public health standards in a growing number of provinces, including Ontario, Nova Scotia, and British Columbia [14-16]. Globally, similar concepts are gaining traction, for example, evidence-informed practice has been acknowledged by the Centers for Disease Control and Prevention as a central component of essential public health services to improve and innovate public health functions [17].

**Figure 1:**
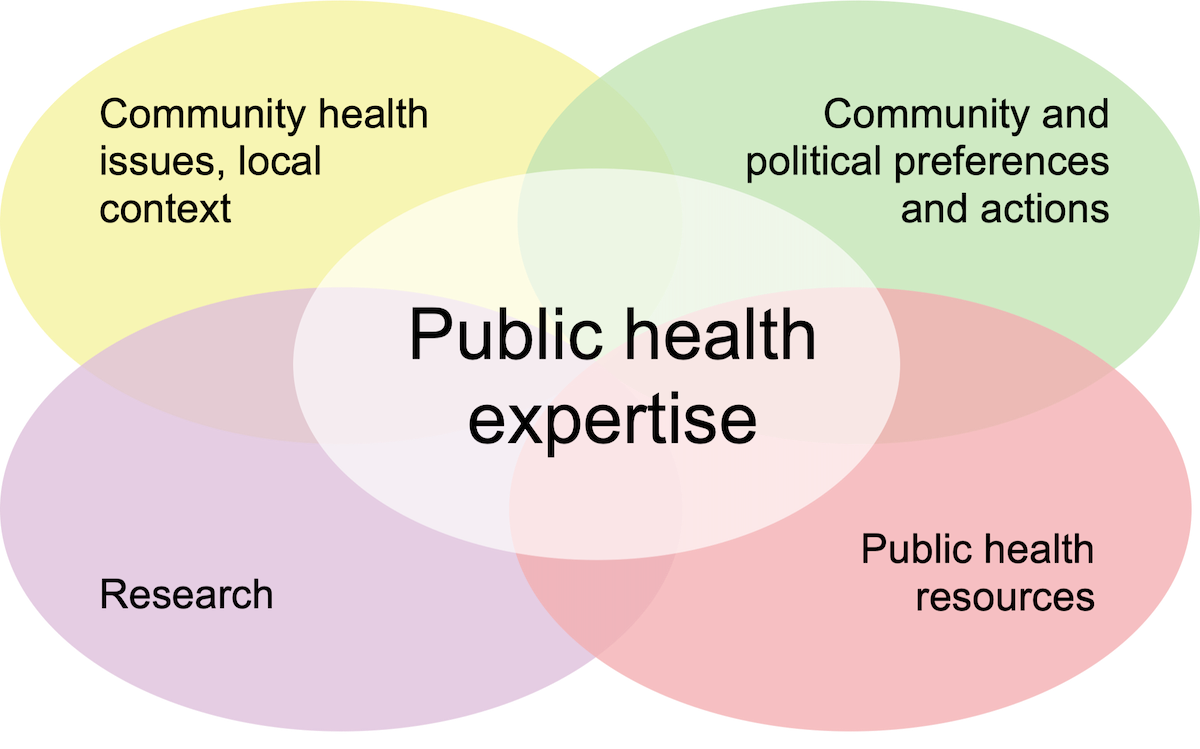
The National Collaborating Centre for Methods and Tools’ evidence-informed decision-making model.

The National Collaborating Centre for Methods and Tools (NCCMT) has developed a model to guide the consideration of different sources of evidence, providing a structure for the use of different types of evidence in the decision-making process (Figure 1) [11]. The 4 spheres of this model are research evidence (published scientific literature, including qualitative or quantitative studies), local context (consideration of the specific needs of the community through quantitative surveillance data, ie, population health indicators), community preferences (using qualitative methods to assess the needs and interests of its members), and resources (human and financial) [11]. Gathering evidence within each of these spheres and making sense of the evidence in relation to a specific jurisdiction is an increasingly daunting task, as the amount of evidence in all spheres grows exponentially [9,18,19]. Previous research has shown that public health professionals value evidence-informed decision-making but encounter barriers such as lack of time; management support; and knowledge and skills to locate, critically analyze, and interpret evidence [9]. Additional challenges exist in appraising, synthesizing, and interpreting different types of evidence, such as limited capacity to apply evidence from the local context and community preferences to program planning [20]. Acquiring and analyzing data to support evidence-informed decision-making can be an intensive process; thus, to truly increase efficiency and effectiveness, system-level support and multiorganization data sharing and computational methods such as artificial intelligence (AI) may offer solutions [9,18,21].

The goal of precision public health is similar to that of evidence-informed decision-making—to put forth effective public health interventions that improve population health [22]. Precision public health is defined as an “emerging practice to more granularly predict and understand public health risks and customize treatments for more specific and homogenous subpopulations, often using new data, technologies and methods” [23]; it aims to improve population health outcomes by enabling the right interventions to be delivered to the right populations at the right time to prevent disease and to protect and promote health [23,24]. Although surveillance systems have traditionally monitored infectious diseases, it is now possible for systems to simultaneously consider data from many sources and apply statistical and AI methods to estimate and monitor the impact of risk factors and diseases on health and other outcomes [21]. AI is a generic term used to define "nonhuman intelligence that is measured by its ability to replicate human mental skills or acting rationally" [25,26]. A hypothetical evidence system that encompasses multiple sources of data and evidence would require the large statistical capabilities of AI to make use of the evidence feasible. It holds promise as a methodological toolbox for supporting public health decision-making and improving population health outcomes, although the evidence is based on a small number of preliminary studies [27,28]. There are many potential uses of AI methods, such as machine learning, in public health, including processing patterns in complex data, modeling policy decisions, and understanding the causal pathways through which interventions influence health outcomes [29]. However, there has been limited implementation of AI in public health initiatives internationally [30]. Although the potential for AI to significantly impact population health exists, substantial human input is required to develop algorithms that can sort and assess evidence inputs and make recommendations for policy and practice [27].

### Objectives

The available evidence systems are limited by the type of evidence they provide, requiring large time and expertise input by professionals to gather and analyze data from multiple platforms [18,31-39]. Currently, there are no public health evidence systems described in the literature that bring together multiple evidence sources in 1 central location with large statistical analysis abilities similar to that of AI; to our knowledge, there is little or no information available on the perceived need for such a system among public health professionals across Canada or internationally. An understanding of the preferences of public health professionals for an electronic evidence system and the desired functionality is critical to inform the development of such systems. The purpose of this study is to identify the needs and preferences of Canadian public health professionals for an electronic evidence system that combines data about local population parameters and context with relevant research evidence about health intervention effectiveness and resources required for successful implementation.

## Methods

### Design

A web-based cross-sectional survey was used to assess the preferences of public health professionals across Canada with respect to an electronic evidence system.

### Study Sample

Eligible participants were individuals currently working in any field in public health organizations in Canada. The web-based survey was available for completion in either English or French. Individuals who identified as students studying public health without any indication of work experience were excluded. Participants were recruited over a 2-week period in February 2019 through the NCCMT’s mailing list (survey was disseminated via email to 11,525 recipients, and 3288 emails were opened) and the Canadian Public Health Association’s bulletin listserv (survey was disseminated via email to 1370 recipients, and 488 emails were opened). Ethics review was not required by the host institution, as this evaluation aimed to inform about the needs for and future development of an electronic evidence system.

### Questionnaire Development

The survey was developed by members of the research team with expertise in public health, AI, and informatics. The survey underwent multiple rounds of consultation between study investigators. Once agreement was reached, the questionnaire was translated by a certified French translator. The final questions were mainly multiple-choice questions, with 1 Likert scale question and 3 open-ended questions.

### Data Collection

Upon initiation of the questionnaire, via LimeSurvey (LimeSurvey GmbH), respondents were asked to consider the following hypothetical scenario:

Imagine an electronic system that combines data about your local population with relevant research evidence about the effectiveness of health interventions. The data in this system would include measures of determinants of health, morbidity, and demographics, and could also be compared to similar measures for other geographic regions / populations. The research evidence could include information on the effectiveness of the interventions in different settings/populations and the resources required for implementing those interventions.

Participants were asked to complete an 18-item questionnaire comprising questions on respondents’ characteristics, preference and need for an electronic evidence system, and barriers and facilitators to use (Multimedia Appendix 1). All responses on LimeSurvey were anonymous, and no identifying data were collected.

### Data Analysis

Quantitative analysis was completed using SPSS (version 25.0, IBM Corp). Descriptive statistics were calculated as means and SDs or percentages, where appropriate. Quantitative responses were categorized post hoc into three types of evidence from the evidence-informed decision-making model: community health issues and local context, research evidence, and public health resources [11]. Given the previous findings that preferences for specific sources of evidence vary by position levels within public health [40,41] and understanding the different perspectives that these groups bring, we planned a subgroup analysis to compare responses by position. We compared the responses of 3 independent categories of positions respondents indicated they held whereas other positions had overlap, as respondents were able to select all position levels that applied. These 3 categories are frontline public health or community providers, project or program management, and senior management or administration. For continuous data, the Levene test was used to assess the homogeneity of variance across the three position groups. Where the assumption of homogeneity was met (*P*=.05), we used a 1-way analysis of variance across the 3 independent groups. When the assumption of homogeneity of variance was not met, the Games-Howell post hoc test was used and the differences among the 3 groups were presented. For categorical data, the Pearson chi-square test was used with comparison across columns. When the cell sizes were less than 5, the Fisher exact test was used to compare the groups.

To analyze the data from the open-ended questions and all other qualitative responses included in the *other* multiple-choice questions open text, data were imported into NVivo (version 12, QSR International). The analysis began with an initial scan of the responses and a discussion of possible themes. Two authors (BD and SENS) independently reviewed the responses using an inductive line-by-line approach and then discussed themes emerging from data and refined the coding scheme [42]. Within the larger theme of needs and preferences, a deductive approach was used where appropriate to code responses according to the following spheres in NCCMT’s evidence-informed decision-making model for public health: community health issues and local context, research evidence, and public health resources [11]. Codes and themes were discussed continuously until the final coding was agreed upon by both the authors.

## Results

### Quantitative Results

A total of 487 respondents clicked on the survey link, initiating the survey. After removing surveys that were not started (n=107) or completed by students (n=9), data from a total of 371 respondents (347 full surveys and 24 partial respondents) were included in this analysis. Respondents were primarily English speakers, with at least a master’s degree, and working in either local, provincial, or territorial government (Table 1). Although many respondents selected multiple positions, frontline public health or community provider (73/371, 19.7%), program or project management (55/371, 14.8%), and senior management or administration (25/371, 6.7%) were largely unique. Respondents reported working in an average of 2.3 (SD 1.8) specific areas of public health, the most commonly being the social determinants of health, chronic diseases, and *all areas* of public health.

**Table 1:** Characteristics of included responses from professionals working in the public health field in February 2019 (N=371).

Characteristics			Respondents, n (%)
**Language**			
	English	361 (97.3)	
	French	10 (2.7)	
**Organization type**			
	Local or regional government	175 (47.2)	
	Provincial government	72 (19.4)	
	University or research center	40 (10.8)	
	Federal government	31 (8.4)	
	Not-for-profit organizations	26 (7)	
	Territorial government	8 (2.2)	
	Indigenous organization	3 (0.8)	
	Consultant organizations	3 (0.8)	
	Primary care or hospitals	2 (0.5)	
	Other or no response	11 (3)	
**Degree**			
	Master’s	206 (55.5)	
	Bachelor’s	96 (25.9)	
	Doctorate	42 (11.3)	
	Diploma	12 (3.2)	
	Doctor of Medicine	11 (3)	
	Other or no response	4 (1.1)	
**Position level**			
	Program or project staff	110 (29.6)	
	Consultant specialist	87 (23.5)	
	Frontline public health or community provider	73 (19.7)	
	Program or project management (eg, manager)	55 (14.8)	
	Faculty	29 (7.8)	
	Senior management or administration (eg, director or executive)	25 (6.7)	
	Government official including policy	21 (5.7)	
	Chief medical or medical or associate medical officer of health	4 (1.1)	
	Other or no response	12 (3.2)	
**Practice discipline**			
	Program evaluator or planner	76 (20.5)	
	Health promoter	74 (19.9)	
	Public health nurse	68 (18.3)	
	Epidemiologist	55 (14.8)	
	Knowledge broker or knowledge translation specialist	52 (14)	
	Health analyst	38 (10.2)	
	Policy analyst	36 (9.7)	
	Administrator or administration	29 (7.8)	
	Policy advisor	24 (6.5)	
	Public health educator	21 (5.7)	
	University or college educator	20 (5.4)	
	Dietitian	18 (4.9)	
	Student	17 (4.6)	
	Librarian or information specialist	13 (3.5)	
	Physician	12 (3.2)	
	Public health inspector	11 (3)	
	Nutritionist	10 (2.7)	
	Other health clinician	10 (2.7)	
	Research staff	6 (1.6)	
	Dentist	3 (0.8)	
	Other or no response	7 (1.9)	
**Area of public health**			
	Social determinants of health	131 (35.3)	
	Chronic disease (eg, nutrition and physical activity)	130 (35)	
	All areas of public health	105 (28.3)	
	Health policy	88 (23.7)	
	Mental health including substance use	85 (22.9)	
	Injury prevention	62 (16.7)	
	Infectious disease	58 (15.6)	
	Family health or reproductive health	54 (14.6)	
	Environmental health	44 (11.9)	
	Reproductive health	29 (7.8)	
	Emergency preparedness or response	25 (6.7)	
	Dental health	17 (4.6)	
	School or child health	11 (3)	
	Hospital care	6 (1.6)	
	Other or no response	9 (2.4)	

The majority of respondents reported that the proposed electronic evidence system would extremely (186/371, 50.1%), very much (141/371, 38%), or moderately (37/371, 9.9%) assist them in their roles. Less than 2% of respondents indicated that an electronic evidence system would only slightly (3/371, 0.8%) or not at all (3/371, 0.8%) help with the work they do. Moreover, 0.3% (1/371) of participants did not answer. Participants’ preferences for community health issues and local contextual data are shown in Table 2. Interest in risk data, namely, prevalence and incidence of disease, was high, along with demographic characteristics. To a lesser degree, respondents reported wanting system functionality to compare their local population with other regions.

**Table 2:** Preferences for community health issues and local context among public health professionals who completed the web-based needs assessment in February 2019 (n=370).

			Respondents, n (%)
**Data**			
	Risk	357 (96.5)	
	Demographics	352 (95.1)	
	Other	107 (28.9)	
**Comparisons**			
	Local to regional	283 (76.5)	
	To smaller subdivisions	283 (76.5)	
	To larger regions	255 (68.9)	
	Other	26 (7)	
**Risk factors**			
	Prevalence	351 (94.9)	
	Incidence	347 (95.1)	
	Other	41 (11.1)	
**Demographics**			
	Age	363 (98.4)	
	Sex	352 (95.1)	
	Income	351 (94.6)	
	Education	336 (90.8)	
	Ethnicity	326 (88.1)	
	Other	98 (26.5)	

A summary of preferences for the types of research evidence is shown in Table 3. Best practice guidelines, systematic reviews or meta-analyses, and practice-based evidence elicited more favorable responses than quantitative or qualitative single studies. Related specifically to interventions, most respondents wanted information about the magnitude of effect and study quality. The required human and financial resources to deliver the intervention and heterogeneity of effects were selected less frequently.

**Table 3:** Preferences for research evidence among public health professionals who completed the web-based needs assessment in February 2019 (n=347).

			Respondents, n (%)
**Types of research evidence**			
	Best practice guidelines		323 (93.1)
	Systematic reviews or meta-analyses		312 (89.9)
	Practice-based evidence		305 (87.9)
	**Single studies**		
		Qualitative	131 (37.8)
		Quantitative	121 (34.9)
	Other		13 (3.7)
**Information about interventions**			
	Magnitude of effect		316 (91.1)
	Quality of study		315 (90.8)
	Required human resources		271 (78.1)
	Required financial resources		261 (75.2)
	Heterogeneity in effect		230 (66.3)
	Other		47 (13.5)

Information about the preference for information about public health resources required is presented in Table 4. The need for information about human resources, including the type and intensity of staff training, training to sustain a program, and the number of staff required, was frequently selected, more so than staff discipline. With respect to financial resources, a preference for cost-effectiveness was most commonly identified, followed by cost. Information on cost-utility and economic modeling were selected less frequently.

**Table 4:** Preferences for information on public health resources among public health professionals who completed the web-based needs assessment in February 2019 (n=347).

			Respondents, n (%)
**Human resources information**			
	Type and intensity of training	295 (85)	
	Type of training to sustain program	277 (79.8)	
	Number of staff required	273 (78.7)	
	Discipline of staff	235 (67.7)	
**Financial resources information**			
	Cost-effectiveness	310 (89.3)	
	Cost	263 (75.8)	
	Cost-utility	160 (46.1)	
	Economic modelling data	124 (35.7)	
	Other	23 (6.6)	

When comparing preferences across the 3 decision-making levels (ie, frontline staff, program management, and senior management), a few notable differences were found (Table 5). Respondents who indicated they were program or project management providers were more likely to indicate a need for demographic data and heterogeneity in effect compared with frontline public health or community providers. No other differences were statistically significant.

**Table 5:** Preferences for an electronic evidence system among frontline public health or community providers, project or program management, and senior management or administration who completed the web-based needs assessment in February 2019.

			Frontline public health or community providers, n/n (%)	Program or project management, n/n (%)	Senior management or administration, n/n (%)
**What data would you want to be included in such a system?**					
	Risk factors		71/73 (97)	52/55 (94)	24/25 (96)
	Demographics		64/73 (88)^a^	54/55 (98)^a^	25/25 (100)
**What would you like to compare your local population with?**					
	Compare your local region with a similar region in size		53/73 (73)	43/55 (78)	21/25 (84)
	Compare subregions within your local regions		59/73 (81)	41/55 (75)	18/25 (72)
	Compare your local region with a larger region in size		50/73 (68)	33/55 (60)	19/25 (76)
**For data related to risk factors and diseases, which data would you want to be included in the system?**					
	Prevalence		69/73 (95)	52/55 (95)	25/25 (100)
	Incidence		68/73 (93)	50/55 (91)	25/25 (100)
**For data related to demographics, which data would you want to be included in the system?**					
	Age		71/73 (97)	54/55 (98)	25/25 (100)
	Sex		68/73 (93)	53/55 (96)	25/25 (100)
	Income		66/73 (90)	53/55 (96)	25/25 (100)
	Education		68/73 (93)	47/55 (85)	22/25 (88)
	Ethnicity		62/73 (85)	51/55 (93)	23/25 (92)
**For research evidence about an intervention, what information would you want to be included?^b^**					
	Magnitude of effect		57/67 (85)	50/53 (94)	22/24 (92)
	Quality of study		55/67 (82)	48/53 (91)	22/24 (92)
	Required human resources		55/67 (82)	42/53 (79)	18/24 (75)
	Required financial resources		52/67 (78)	39/53 (74)	18/24 (75)
	Heterogeneity in effect		36/67 (54)^c^	40/53 (75)^c^	15/24 (62)
**Which of the following research evidence options would you want to be made available?^b^**					
	Best practice guidelines		64/67 (95)	50/53 (94)	23/24 (96)
	Systematic reviews or meta-analyses		53/67 (79)	45/53 (85)	19/24 (79)
	Practice-based evidence (program evaluations)		56/67 (84)	51/53 (96)	20/24 (83)
	**Single studies**				
		Qualitative	27/67 (40)	21/53 (40)	6/24 (25)
		Quantitative	24/67 (36)	18/53 (34)	7/24 (29)
**For human resources, which information would you want available from the evidence?^b^**					
	Type and intensity of training required to be competent to deliver interventions or programs		23/67 (96)	49/53 (92)	19/24 (79)
	Type of training required to sustain program		19/67 (79)	41/53 (77)	21/24 (87)
	Number of staff required to implement the program		20/67 (83)	47/53 (89)	20/24 (83)
	Discipline of required staff		6/67 (25)^a^	40/53 (75)	19/24 (79)^a^
**For financial resources, which information would you want available?^b^**					
	Cost-effectiveness		61/67 (91)	47/53 (89)	21/24 (87)
	Cost		47/67 (70)	41/53 (77)	19/24 (79)
	Cost-utility		28/67 (42)	29/53 (55)	12/24 (50)
	Economic modeling data		23/67 (34)	20/53 (38)	10/24 (42)

^a^Indicates statistically significant difference (*P*=.04).

^b^Some participants only provided partial answers to the survey; thus, the sample sizes differ across questions.

^c^Indicates statistically significant difference (*P*=.045).

### Qualitative Results

Qualitative data from open-ended questions identified several specific needs, concerns, and suggestions for an electronic evidence system. Echoing the preferences for cross-jurisdictional comparisons found in the quantitative results, respondents identified the ability to compare indicators across geographic areas, the inclusion of equity indicators and epidemiologic data, and the use of geographic information systems as *other* specific requests. Health equity indicators, such as the determinants of health, were seen as important in identifying and describing vulnerable populations. One respondent stated:

...generally, any data that might link to poverty measures, immigration status, housing situation (e.g., housed, homeless), recipient of childcare subsidy, recipient of social assistance etc.

A major theme that emerged with respect to the type of research evidence to be included was the usefulness of research beyond what is typically considered public health interventions, such as organizational interventions and interventions from the fields of education, social services, and law. Regardless of the type of research, there was a strong desire for all evidence to be critically appraised and be presented alongside summaries or statements to help interpret the evidence, as illustrated in the following quote:

...while I would be open to including all kinds of research, I would want them to be graded, to ensure that one could assess the quality of the evidence.

Similarly, participants also emphasized the need for practice-based evidence that provides contextual information on the outcomes of interventions and implementation. This included evidence on the context in which an intervention was implemented, adoption of the intervention, and considerations on how to deliver and sustain it in the community. This is reflected in a respondent’s comment:

[I] need a way to analyze context where an intervention is used. For example, if previously similar interventions had been tried in an area or subpopulation there may already be a delivery system or key partnerships in place, and there may also be a learning effect from previous work that is beneficial to achieving results with a “new” intervention.

To support the need for contextual and implementation data, respondents also specifically mentioned the need for qualitative and mixed-methods research and needs assessments conducted within other communities or organizations.

Related to resources and tools for practice, a need for theories, methods, or frameworks to support adaption or to implement a program in their community was identified. Some respondents mentioned specific frameworks, such as the Reach, Effectiveness, Adoption, Implementation, and Maintenance framework, whereas others had general suggestions for *evaluation* or *implementation* frameworks. There were also requests for tools to support practice, such as the Applicability and Transferability Tool [43], which supports public health planners’ use of evidence to support appropriate programming for the community, or survey question templates.

In addition to the specific needs for an electronic system, a number of potential concerns or barriers emerged. Concerns were related to either the electronic system itself or the ability to adopt a system within public health organizations. Concerns about keeping a system up to date stemmed from the understanding that evidence is created at rapid rates and new data are constantly being collected. For such a system to be useful, data would need to be current. Sustainability of the system beyond its initial creation was seen as a critical element for successful implementation, with some participants citing concerns if the system were to be funded by a research grant. An understanding of plans for long-term upkeep and sustainability may be a requirement for individual users or organizations to invest time in learning how to use the system.

The potential for duplication of existing resources was another concern related to such a system, with respondents citing specific databases or systems that already exist, and how existing databases and systems would complement or conflict with any new system. One respondent captured this sentiment, stating that:

...these systems are difficult to set up AND keep up to date. In addition, other similar systems (except for intervention data) already exist and this may add to the confusion for users (which data is THE official data?) Why do we observe differences between two systems for same indicator? Etc.

Related to the ability of individuals and organizations to adopt and implement the system, major themes about usability and costs emerged. The cost of the proposed system was seen as a key potential barrier, with questions about who would pay for it arising frequently. Second, the ability of a system to work with existing information technology infrastructure, such as outdated or restrictive computer systems and limited or slow internet connectivity, was raised as a concern. Beyond the initial barriers of cost and access, an organization’s ability to adopt the use of a system in their regular workflow was reported to be dependent on the ability of individual staff to use the system adequately, which requires not only buy-in by the individual employee but also senior-level management. Finally, concerns about data privacy and maintenance of confidentiality were also expressed.

A number of suggestions for success emerged from the qualitative data. The most frequently mentioned requirement to facilitate use of the system were transparency of methodology used, including the criteria to select evidence for inclusion, the methods used to evaluate and synthesize evidence, and the overall quality of the evidence included. One respondent stated that they “...would need a very detailed ‘methods’ section of this system to be able to be confident in it.” Sufficient staff training was also suggested to support the use of the proposed system.

Finally, respondents requested specific functions or system formatting elements, such as the ability to make graphs, print or export data, and retrieve contact information of data sharers on the system.

## Discussion

### Principal Findings

The purpose of this study is to understand the preferences of Canadian public health professionals for an electronic evidence system. The results indicate that there is a perceived need for an electronic evidence system; however, certain considerations related to the type of information included and how it would be presented must be addressed for such a system to be adopted and used effectively for public health decision-making.

Preferences for all 3 types of evidence (community health issues and local context, research evidence, and public health resources) were generally high. This aligns with previous research that public health professions value different sources of evidence [20]. An important consideration to emerge from both the quantitative and qualitative data was the need to understand the quality of the evidence included within the system. Participants suggested that the evidence included in an electronic evidence system should be preappraised and include a statement of interpretation along with a description of the methodology used to appraise the evidence. Using the best available evidence is a critical component of evidence-informed decision-making [11]. Critical appraisal requires knowledge and skill development through training and time to appraise evidence on a continual basis. Respondents were aware that there was a need for evidence to be appraised but wanted a system to do this for them. This was also the case for the ability to interpret evidence appropriately, and there was recognition that there may be various levels of skills to understand evidence. These findings are in line with previous literature that shows that time, knowledge, and skills in appraising different types of evidence are a barrier to evidence-informed decision-making in public health [9,20]. A qualitative study involving public health decision makers found that clear implication statements from the evidence facilitated uptake of this knowledge in practice and decision-making processes [44]. Including evidence that has been preappraised and accompanied by interpretation statements, possibly through AI approaches within an electronic evidence system, may make it easier for users to understand and use the evidence, effectively overcoming some challenges to the evidence-informed decision-making process.

The need for information to examine and address the determinants of health and health equity came through strongly in this study. This is not surprising given the previous literature that suggests that equity information is commonly lacking in scientific publications. A 2016 scoping review of population health interventions found that most studies included minimal contextual information on the target population and intervention setting [19]. This contextual information is important for effective decision-making, as it is necessary to appropriately apply evidence in different settings. Furthermore, concerns have been raised about the potential of AI to "amplify inequities in society" because of inherent biases in data sets and programming on a large scale [45]. Although AI is useful in identifying which trends are occurring, some AI methods, such as machine learning, may lack the ability to describe why the pattern occurs [46]. An understanding of contextual indicators such as the social determinants of health can improve the adoption and sustainability of public health investment and potentially limit biases embedded within an electronic evidence system [19,45,46].

A key concern that emerged from the qualitative data was avoiding duplication of existing resources, some of which were already in use within their organization. For example, in Canada, the Canadian Best Practice Portal captures intervention evidence on effective health promotion and chronic disease prevention, but it is no longer updated [31]; OpenData shares surveillance evidence nationally and its uses in practice [33]; Statistics Canada provides access to census-based population data [34]; and Health Evidence provides quality assessments of systematic reviews of public health interventions [32]. However, these are independent platforms that search for and synthesize data and do not integrate different types of evidence, such as local context and public health resources, requiring users to search multiple platforms [18]. There have been calls to action from experts in public health and health informatics for pan-Canadian collaborative efforts to facilitate access to databases across the country [29]. Until then, any new electronic evidence system should explore partnerships with relevant existing platforms and mechanisms to avoid duplication of resources and efforts.

Barriers to the use of an electronic evidence system identified in this survey are similar to those found in a previous systematic review on barriers to public health data sharing [47]. In the review, the authors identified six main categories of barriers: technical, motivational, economic, political, legal, and ethical [47]. Regarding technical barriers, concerns about the integration of a new electronic evidence system within the existing information technology infrastructure of an organization emerged from the qualitative data [47]. Economic barriers related to initial and ongoing financial costs were also raised [47]. Some participants in this study expressed concerns with respect to legal barriers, such as data privacy and confidentiality. Both technical and economic barriers illustrate the need for greater organizational capacity development [47]. A previous review suggested allocating 5%-10% of program funds to data collection, monitoring, evaluation, and operational research, while recognizing that larger systems change needs to occur simultaneously to build sustainable funding mechanisms [47]. Future research is needed to further understand how best to implement such a system in a way that overcomes the known technological barriers such as interoperability and cost, among others.

In our survey, motivational, political, and ethical barriers were not raised; however, the survey did not specifically seek feedback on these factors. Although motivational barriers, which limit data sharing at an individual or organizational level, were not explicitly mentioned, some respondents suggested possible ways to overcome a component of this barrier, disagreements in data use [47]. Respondents suggested providing contact information of researchers who shared the data or the inclusion of a networking component in the electronic evidence system to facilitate discussion about the data, implementation of the possible intervention, successes or failures of interventions in different contexts, etc. The ability to have discussions between the data donor and the researcher using the data may increase trust between both parties, transparency, and reliability of the platform. As mentioned in the 2014 review, the 6 categories of barriers have complex interactions, which need to be addressed with a comprehensive approach to ensure usability of a potential electronic evidence system [47].

An additional barrier identified in the qualitative responses was the need for ongoing training of staff to use the system. Although AI has the potential to compile, process, synthesize, and analyze patterns at rapid rates and to improve efficacy in the use of evidence, blind reliance on its outputs runs the risk of misrepresenting variables or groups of people as it is dependent on data collection methods and evidence inputs [29,46,48]. Experts recommend that AI-specific training is also needed if users are to appropriately address concerns of equity and systemic biases in electronic evidence systems [29,46,48]. This highlights an important consideration for future implementation of such a system. A potential avenue for training can be through web-based learning modules, as they have been found to be effective for public health professionals in one study [49]. Web-based modules that provide training on how to optimize the system and offer other features suggested by respondents, including videos, webinars with creators of the system, and social networking features to connect with other users and researchers, may support public health professionals in using the system efficiently and overcome the aforementioned barriers.

### Limitations

There are some limitations to this study, which should be considered when interpreting the findings. First, we did not collect any individual demographic data or years of experience working in public health, limiting the extent to which we can characterize the types of individuals who took part in this survey. In allowing participants to select all that apply for organization type, role, area of public health, and practice discipline, our analysis was limited in its ability to compare differences in preferences across each of these categories. Although the survey was disseminated through two large Canadian-based listservs to recruit public health professionals, there was no qualifying question to confirm that the preferences that emerged were solely of public health professionals in Canada. Second, respondents in the survey ranged across roles, areas, and disciplines, and their results may not be generalizable across all Canadian public health professionals, as respondents who participated may have prior awareness of, or an interest in, electronic evidence systems or evidence-informed decision-making. Finally, the survey questions for a hypothetical electronic evidence system without considerations of feasibility may have skewed responses positively, where respondents more favorably indicated the need for all items listed [50].

### Conclusions

Public health professionals and organizations face many hurdles, including changes in structure, lack of funding and time, and exponential increases in new evidence. However, there is broad agreement that the hypothetical electronic evidence system proposed would make informed decisions more accessible. On the basis of our findings, public health professionals see the value in an electronic evidence system that combines local contextual evidence, research and intervention studies, and public health resources and tools. Our findings also highlight a number of elements that should be considered to ensure usability and facilitate trust in such an electronic evidence system. These elements include quality appraisals, interpretations of evidence, and transparent methods and funding models. Such an electronic evidence system may support professionals in evidence-informed decision-making, thereby enabling the Canadian public health system to be more effective in an environment with limited investment.

## Appendix

Multimedia Appendix 1 Categorized needs assessment questionnaire items and answer options.

## Abbreviations

AI: artificial intelligence
NCCMT: National Collaborating Centre for Methods and Tools

## References

[1] (2018). Structural profile of public health in Canada. National Collaborating Centre for Healthy Public Policy.

[2] Fedeli V (2019). 2019 Ontario Budget: Protecting What Matters Most.

[3] Guyon A, Perreault R (2016). Public health systems under attack in Canada: evidence on public health system performance challenges arbitrary reform. Can J Public Health.

[4] (2019). Alberta Health Services performance review. Government of Alberta.

[5] Himmelstein DU, Woolhandler S (2016). Public health’s falling share of US health spending. Am J Public Health.

[6] (2019). PHE annual report and accounts: 2018 to 2019. Public Health England.

[7] Maani N, Galea S (2020). COVID-19 and underinvestment in the public health infrastructure of the United States. Milbank Q.

[8] Murray CJ, Alamro NM, Hwang H, Lee U (2020). Digital public health and COVID-19. Lancet Pub Health.

[9] Brownson RC, Fielding JE, Green LW (2017). Building capacity for evidence-based public health: reconciling the pulls of practice and the push of research. Annu Rev Public Health.

[10] Kohatsu N, Robinson J, Torner J (2004). Evidence-based public health: an evolving concept. Am J Prev Med.

[11] (2009). A model for evidence-informed decision making in public health. National Collaborating Centre for Methods and Tools.

[12] Yost J, Dobbins M, Traynor R, DeCorby K, Workentine S, Greco L (2014). Tools to support evidence-informed public health decision making. BMC Public Health.

[13] Culyer AJ, Lomas J (2006). Deliberative processes and evidence-informed decision making in healthcare: do they work and how might we know?. Evid Policy.

[14] (2018). Ontario public health standards: requirements for programs, services, and accountability. Ministry of Health and Long-Term Care, Government of Ontario.

[15] (2016). Nova Scotia public health standards 2011-2016. Government of Nova Scotia.

[16] (2016). Public health economics. Ministry of Health, British Columbia.

[17] (2020). The 10 essential public health services. Centers for Disease Control and Prevention.

[18] Shaban-Nejad A, Lavigne M, Okhmatovskaia A, Buckeridge D (2017). PopHR: a knowledge-based platform to support integration, analysis, and visualization of population health data. Ann N Y Acad Sci.

[19] Shoveller J, Viehbeck S, Di Ruggiero E, Greyson D, Thomson K, Knight R (2015). A critical examination of representations of context within research on population health interventions. Critical Public Health.

[20] Martin W, Higgins JW, Pauly B, MacDonald M (2017). "Layers of translation" - evidence literacy in public health practice: a qualitative secondary analysis. BMC Public Health.

[21] Groseclose SL, Buckeridge DL (2017). Public health surveillance systems: recent advances in their use and evaluation. Annu Rev Public Health.

[22] Dobbins M, Buckeridge D (2020). Precision public health: dream or reality?. Can Commun Dis Rep.

[23] Dolley S (2018). Big data's role in precision public health. Front Public Health.

[24] Buckeridge DL (2020). Precision, equity, and public health and epidemiology informatics - a scoping review. Yearb Med Inform.

[25] De Spiegeleire S, Maas M, Sweijs T (2017). Artificial intelligence and the future of defense: strategic implications for small- and medium-sized force provider.

[26] Russell S, Norvig P, Davis E (2010). Artificial Intelligence: A Modern Approach.

[27] Cresswell K, Callaghan M, Khan S, Sheikh Z, Mozaffar H, Sheikh A (2020). Investigating the use of data-driven artificial intelligence in computerised decision support systems for health and social care: a systematic review. Health Informatics J.

[28] Triantafyllidis AK, Tsanas A (2019). Applications of machine learning in real-life digital health interventions: review of the literature. J Med Internet Res.

[29] (2018). Application of artificial intelligence approaches to tackle public health challenges - workshop report. Canadian Institutes of Health Research.

[30] Morgenstern JD, Rosella LC, Daley MJ, Goel V, Schünemann Holger J, Piggott T (2021). "AI's gonna have an impact on everything in society, so it has to have an impact on public health": a fundamental qualitative descriptive study of the implications of artificial intelligence for public health. BMC Public Health.

[31] (2014). Canadian best practices portal. Government of Canada.

[32] Helping public health use best evidence in practice since 2005. Health Evidence.

[33] Open data. Government of Canada.

[34] Data. Statistics Canada.

[35] HealthLandscape.

[36] CDC Wonder. Centers for Disease Control and Prevention.

[37] Behavioral risk factor surveillance system. Centers for Disease Control and Prevention.

[38] Database of promoting health effectiveness reviews (DoPHER). EPPI-Centre.

[39] (2021). National Center for Health Statistics. Centers for Disease Control and Prevention.

[40] Peirson L, Ciliska D, Dobbins M, Mowat D (2012). Building capacity for evidence informed decision making in public health: a case study of organizational change. BMC Public Health.

[41] Dobbins M, Traynor RL, Workentine S, Yousefi-Nooraie R, Yost J (2018). Impact of an organization-wide knowledge translation strategy to support evidence-informed public health decision making. BMC Public Health.

[42] Azungah T (2018). Qualitative research: deductive and inductive approaches to data analysis. Qual Res J.

[43] Buffet C, Ciliska D, Thomas H (2007). Can I use this evidence in my program decision? Assessing applicability and transferability of evidence. National Collaborating Centre for Methods and Tools.

[44] Dobbins M, Jack S, Thomas H, Kothari A (2007). Public health decision-makers' informational needs and preferences for receiving research evidence. Worldviews Evid Based Nurs.

[45] Panch T, Mattie H, Atun R (2019). Artificial intelligence and algorithmic bias: implications for health systems. J Glob Health.

[46] (2019). AI for public health equity - workshop report. Canadian Institutes of Health Research.

[47] van Panhuis WG, Paul P, Emerson C, Grefenstette J, Wilder R, Herbst AJ, Heymann D, Burke DS (2014). A systematic review of barriers to data sharing in public health. BMC Public Health.

[48] Panch T, Pearson-Stuttard J, Greaves F, Atun R (2019). Artificial intelligence: opportunities and risks for public health. Lancet Dig Health.

[49] Kenefick HW, Ravid S, MacVarish K, Tsoi J, Weill K, Faye E, Fidler A (2014). On your time: online training for the public health workforce. Health Promot Pract.

[50] Lavrakas P (2008). Encyclopedia of Survey Research Methods.

