# Supplementary material for: Assessing the Electronic Evidence System Needs of Canadian Public Health Professionals: Cross-sectional Study
Source: JMIR Public Health Surveill. 2021 Sep 7;7(9):e26503. doi: 10.2196/26503 (PMC8456326; doi:10.2196/26503)
Supplement: Multimedia Appendix 1 [file publichealth_v7i9e26503_app1.pdf]

Additional file 1. Categorized needs assessment questionnaire items and answer options

Category	Question	Options
Characteristics	Which organization type listed below do you work at? (Choose one of the following answers)	• Local or regional government/paragovernmental<br>• Provincial government/paragovernmental<br>• Territorial government/paragovernmental<br>• Federal government/paragovernmental<br>• Not-for-profit organization, non-governmental, or foundation<br>• University or Research centre<br>• Indigenous organisation<br>• Other (please specify)
	What is your highest degree earned? (Choose one of the following answers)	• Diploma<br>• Bachelors<br>• Masters<br>• Doctor of Medicine<br>• Doctorate<br>• Other (please specify):
	Which of the following describes your practice discipline? (Check ALL that apply)	• Administrator/Administration<br>• Dietitian<br>• Nutritionist<br>• Epidemiologist<br>• Health Analyst<br>• Health Promoter<br>• Public Health Inspector<br>• Knowledge Broker/Knowledge Translation Specialist<br>• Librarian/Information Specialist<br>• Public Health Nurse<br>• Physician<br>• Dentist<br>• Other Health Clinician<br>• Policy Analyst<br>• Policy Advisor<br>• Program Evaluator/Planner<br>• Public Health Educator<br>• University/College Educator<br>• Student<br>• Other (please specify):
	Which of the following describes your position level? (Check ALL that apply)	• Administration Assistant<br>• Front-Line Public Health/Community Provider<br>• Program/Project staff<br>• Consultant Specialist<br>• Program/Project Management (e.g., Manager)<br>• Faculty<br>• Senior Management/Administration (e.g., Director, Executive)<br>• Chief Medical Officer of Health/Medical Officer of Health/Associate Medical Officer of Health

		• Government Official (policy)<br>• Other (please specify):
	Which of the following describes your practice area in public health? (Check ALL that apply)	• All Areas of Public Health<br>• Environmental Health<br>• Chronic Disease (e.g., nutrition, physical activity)<br>• Injury Prevention<br>• Family Health/Reproductive Health<br>• Infectious Disease<br>• Emergency Preparedness/Response<br>• Mental Health (including substance use)<br>• Dental Health<br>• Reproductive Health<br>• Social Determinants of Health<br>• Health Policy<br>• Other (please specify):
Overall need	To what extent would an electronic system that combines data and research evidence for the purpose of identifying evidence-based policies and programs assist in decision making for service delivery? (5 point Likert scale)	• Would **not at all** assist in decision making<br>• Would **slightly** assist in decision making<br>• Would **moderately** assist in decision making<br>• Would **very much** assist in decision making<br>• Would **extremely** assist in decision making
Community health issues, local context sphere	What data would you want included in such a system? (Check ALL that apply)	• Risk factors and diseases (e.g., incidence, prevalence)<br>• Demographics (e.g., population counts, mortality)<br>• Other (please specify)
	Would it be helpful to compare data for your local population to data for other geographic regions? (Check ALL that apply)	• Compare my local population to other regions at the same level of geographic resolution (e.g. province to other provinces)<br>• Compare my local population to larger regions (e.g. health region to the enclosing province or country)<br>• Compare smaller subdivisions within my region (e.g. health regions within a province)<br>• Other (please specify)
	For data related to risk factors and diseases which data would you want to be included in the system? (Check ALL that apply)	• Incidence<br>• Prevalence<br>• Other (please specify)
	For data related to demographics which data would you want to be included in the system? (Check ALL that apply)	• Age<br>• Sex<br>• Ethnicity<br>• Income<br>• Education<br>• Other (please specify)
Research evidence sphere	For research evidence about an intervention, what information would you want to be included? (Check ALL that apply)	• Magnitude of effect<br>• Heterogeneity in effect<br>• Required human resources<br>• Required financial resources<br>• Quality of study<br>• Other (please specify)
	Which of the following research evidence options would you want to be	• Best practice guidelines<br>• Systematic reviews/meta-analyses

	made available? (Check ALL that apply)	• Single studies (quantitative)<br>• Single studies (qualitative)<br>• Practice-based evidence (program evaluations)<br>• Other (please specify)
Public health resources sphere	For human resources, which information would you want available from the evidence? (Check ALL that apply)	• Discipline of required staff<br>• Numbed of staff required to implement the intervention/program<br>• Type and intensity of training required to be competent to deliver interventions/programs<br>• Type of training required to sustain program
	For financial resources, which information would you want available to be provided? (Check ALL that apply)	• Costs<br>• Cost-effectiveness<br>• Cost-utility<br>• Economic modelling data<br>• Other (please specify)
Open-ended questions	Are there forms of evidence or information you would like included in an electronic evidence and information system?	
	Is there anything else you would want to know about this system in order to be comfortable and confident using it?	
	Do you foresee any barriers to using an electronic evidence and information system?	
